# Supplementary material for: May the force be with you: The role of hyper-mechanostability of the bone sialoprotein binding protein during early stages of Staphylococci infections
Source: Front Chem. 2023 Feb 8;11:1107427. doi: 10.3389/fchem.2023.1107427 (PMC9944720; doi:10.3389/fchem.2023.1107427)
Supplement: Supplementary file 1 [file DataSheet1.PDF]

May the force be with you: the role of hyper-mechanostability of the bone sialoprotein binding protein during early stages of *Staphylococci* infections.

Priscila S. F. C. Gomes [1], Meredith Forrester [1], Margaret Pace [1], Diego E. B. Gomes [1] and Rafael C. Bernardi [1,*]

[1] *Department of Physics, College of Sciences and Mathematics, Auburn University, Auburn, AL, 36849*

Correspondence*:
Rafael C. Bernardi
rcbernardi@auburn.edu

SUPPLEMENTARY INFORMATION

Table S1. Pulling speeds and number of replicas used for the different systems and MD protocols.

Protocol	Pulling speed (nm/ps)	Replicas - original	Replicas - elongated Fgα
aa-SMD	2.5×10^{-01}	192	-
aa-SMD	2.5×10^{-02}	192	192
aa-SMD	2.5×10^{-03}	160	192
aa-SMD	1.25×10^{-03}	160	-
aa-SMD	2.5×10^{-04}	160	192
aa-SMD	2.5×10^{-05}	-	96
CG-SMD	2.5×10^{-03}	128	-
CG-SMD	2.5×10^{-04}	128	512
CG-SMD	2.5×10^{-05}	64	512
CG-SMD	2.5×10^{-06}	64	128

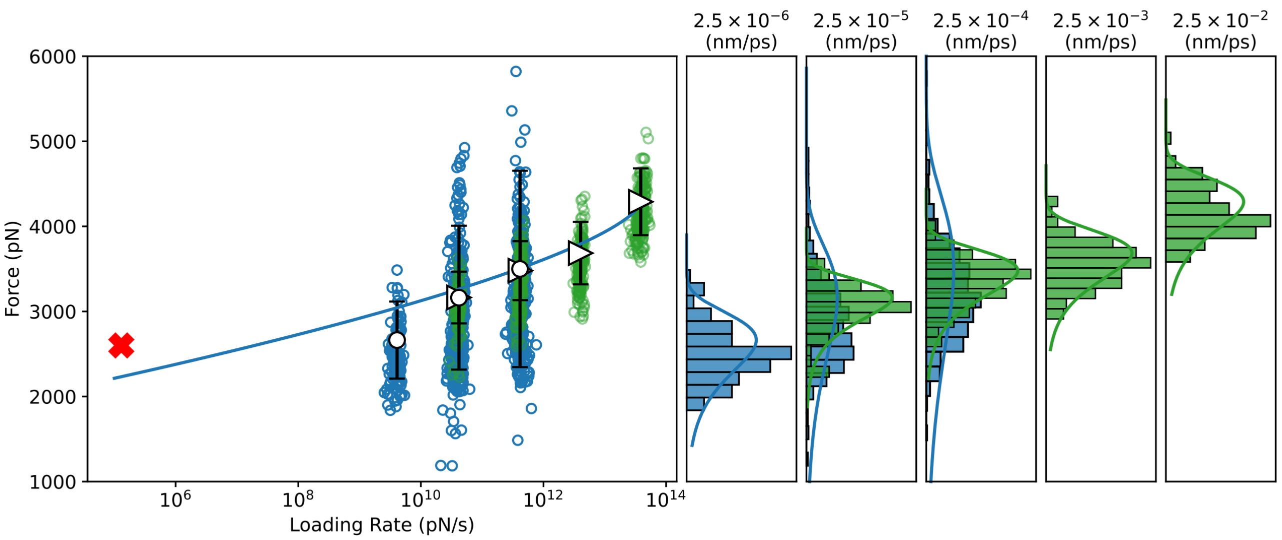

Figure S1. Dynamic Force spectrum for the BBP complexed with the longer Fgα peptide combining data from all-atom and coarse-grained SMD simulations. All-atom, and Coarse-grained steered molecular dynamics simulations (CG-SMD and aa-SMD) were performed at different velocities: 2.5×10^{-5} to 2.5×10^{-2} nm/ps (green) and 2.5×10^{-6} to 2.5×10^{-4} nm/ps (blue), respectively. A Dudko-Hummer-Szabo **?** (DHS) fit was performed through the SMD dataset predicting $\Delta x = 8.584 \times 10^{-2}\ nm$, $k_{off}{}^{0} = 9.365 \times 10^{-15}\ s^{-1}$, $\Delta G = 2.449 \times 10^{2}\ k_B T$.

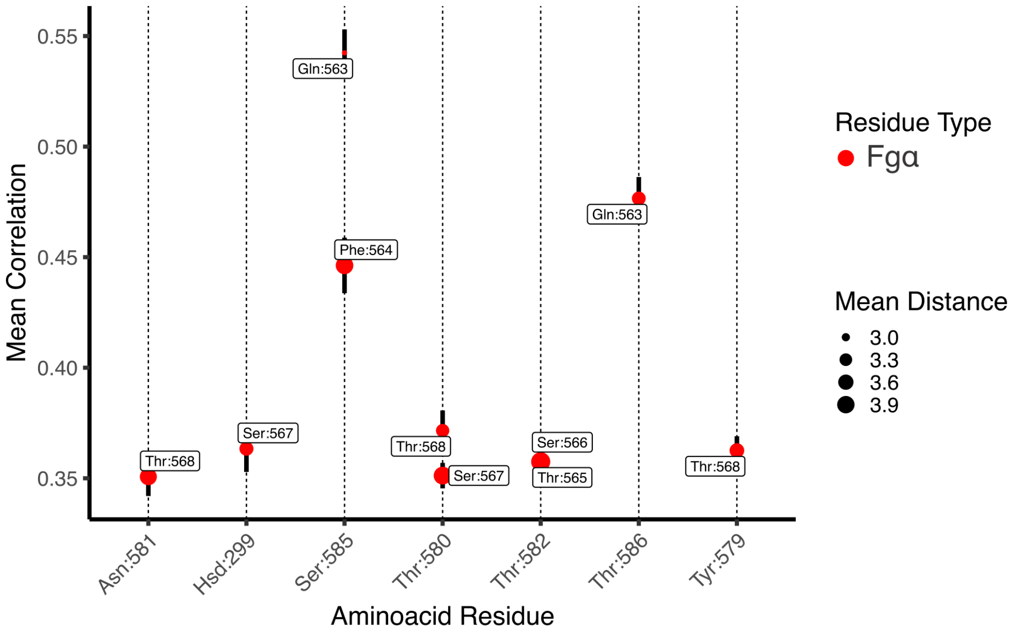

Figure S2. Mean generalized coefficients for contacts along the interface between Bbp complexed with the longer Fgα peptide. The x axis is labeled by Bbp amino acid residues and the y axis indicates the averaged generalized correlation values (vertical bars indicate the standard error of the mean), labeled by Fgα aminoacid residues. The circle sizes indicates the average Cartesian distance. Only amino acid residues with a mean correlation higher than 0.35 are shown.
